# Glances at the Insane in Other Lands

**Published:** 1889-03-16

**Authors:** 


### Glances at the Insane in Other Lands.

By a Medical Superintendent.

*(Continued from p. 369.)*

### VII.—ALABAMA.

The State of Alabama has an area of upwards of fifty thousand square miles, being nearly as large as England and Wales. It is wedged between Georgia on the one hand, and Mississippi on the other. To the north is Tennessee, and to the south Florida; but there is a little bit of freeboard to the Gulf of Mexico. On this stands the town of Mobile, famous as a great cotton mart, and as one of the blockaded ports in the days of the Civil War. The population of the State is over a million, and a very considerable proportion, probably more than one-third, is coloured.

The asylum is situated near Tuscaloosa, which used to be the seat of government until Montgomery was raised to that distinction. We notice that many of the American asylums are verging on the highest number of patients that can be reasonably well looked after under one roof, and some of them, the Alabama one among others, have crossed the boundary. It contains eleven hundred beds, and there are nearly one thousand patients in residence. It is anticipated that in a year or so all the beds will be occupied, and when this point is reached, patients can only be admitted when vacancies arise from death or discharges. But then, vacancies thus created will not be nearly sufficient to meet the demands, and the medical superintendent proposes to have a law enacted whereby harmless cases may be discharged to their homes. In England we have such a law, and although something may be done in this way, all the trials made on this side of the Atlantic have shown that the relief obtained by these means is either slight or temporary; very often it is both slight and temporary. That any considerable enlargement of an asylum already containing a thousand beds is desirable, cannot be thought of, and there is only one way out of the difficulty: that is, erecting another hospital at some convenient point of the State. Dr. Bryce says: "There are many advantages in having these institutions for the treatment and care of the insane at different points in the state. Massachusetts has eight public asylums located in different sections of the State; New York has ten, and as many more county and private asylums; Pennsylvania has six; Ohio has seven. Virginia has five; Kentucky has three; Texas has three; and our neighbour, Mississippi, has two."

The trustees think that some action ought to be taken by the General Assembly for the separation of criminal lunatics from the others; but in the meantime they would be satisfied if previous notice were sent to the asylum of the intention to remove the criminal patient. "As it is," the trustees state, "they are dumped down on us by the sheriffs, under order of the Courts, without proper notice, and without opportunity to prepare for their custody, and without regard to the question of there being any room for them in the hospital." The truth is, that criminal lunatics ought never to be treated in the same building with the ordinary insane.

The medical superintendent's report is biennial only; a system not uncommon in America. On September 30th, 1886, there were 733 patients in the asylum, and on September 30th, 1888, the numbers had risen to 957. Of these 772 are white; 185 coloured; 33 are private patients, and 927 are State-supported. During the two years 113 patients died, showing a low mortality. Judging from our perusal of American asylum reports, we infer that phthisis is commoner in the States than in England. At the Tuscaloosa asylum 29 of the 113 deaths were owing to it. In nine cases death was caused by cerebral hæmorrhage—a disease which is not very often met with among our insane. As to the recoveries, Dr. Bryce thinks that anywhere between 25 and 40 per cent. of the admissions would represent the proportion. In 1886 the percentage was 40, and in 1888 it fell to 22. A good deal depends on the standard of mental soundness employed; and when talking about the recoveries, "we should always remember the re-admissions."

In the entire abolition of mechanical restraint the Alabama asylum is well to the front; and the open-door system is also in use with the best results. "We can now open our ward doors and allow a large number of our patients to go in and out at pleasure, without the least apprehension that such privilege will be abused." It is really very gratifying to read the above sentence, because in England we are rather apt to think that, as some American asylums indulge in restraint all do it.

The employment of the patients is so carefully attended to that 90 per cent. of the women and 60 per cent. of the men do work of some kind.

The cost per head per week has been reduced from 10s. 6d. to 9s. 5d., a rate which is certainly below the average of American asylums.

It struck us that the statistical tables are somewhat meagre in their supply of information.

There only two assistant physicians. Dr. Bryce oils the backs of his officers, and the trustees oil the doctor's back. They are apparently quite a happy family. We have read their reports with pleasure, and we hope with profit, which is more than we can always say of similar documents.

